# Body Weight Development in Adult Dogs Fed a High Level Resistant Starch Diet

**DOI:** 10.3390/ani12233440

**Published:** 2022-12-06

**Authors:** Kangmin Seo, Hyun-Woo Cho, Ju Lan Chun, Kyoung Min So, Ki Hyun Kim

**Affiliations:** 1 Animal Welfare Research Team, National Institute of Animal Science, Rural Development Administration, Wanju 55365, Republic of Korea; 2 Division of Animal Disease and Health, National Institute of Animal Science, Rural Development Administration, Wanju 55365, Republic of Korea

**Keywords:** dog, obesity, liver function, resistant starch, ALT, pet food

## Abstract

**Simple Summary:**

As companion animals are incorporated further into the family unit, interest in their health and longevity is increasing. Globally, the estimated obesity rate of companion animals is ~60%, and concerns over obesity-related diseases and quality of life are rising. Obesity in companion animals, much like in humans, negatively affects their health due to diseases such as diabetes, cardiovascular disease, joint disease, and abnormal lipid metabolism. In addition to increasing the burden of care costs on pet owners, this lowers the quality of life for companions and pet owners alike. Recently, resistant starch has been spotlighted as an excellent ingredient for obesity prevention and weight loss due to its indigestible properties in humans. We evaluated the feeding effect of pet food high in resistant starch in dogs, such as Dodamssal rice. The results of this study suggested that Dodamssal rice can be used as a raw ingredient for the prevention of obesity in dogs.

**Abstract:**

This study investigated the effect of Dodamssal rice, which has a high content of resistant starch, on obesity and hematologic properties in dogs. In Experiment 1, 24 spayed dogs were divided into three feeding groups: normal-fat basal diet (control), high-fat diet with 12% normal amylose type rice (hNAR), and high-fat diet with 12% high amylose type rice (Dodamssal rice; hHAR). In Experiment 2, 8 spayed dogs were assigned to a normal amylose type rice (NAR) group and a high amylose type rice group (HAR) with a normal-fat basal diet. After 24 weeks, an increase in weight and blood cholesterol was observed in both high-fat diet groups for Experiment 1. Specifically, an increase in serum alanine aminotransferase was observed over time in the hNAR group compared with that of the control; however, no such patterns were present in the hHAR group. Further, a significant weight-loss effect was observed in the HAR group in Experiment 2 at 4 weeks. The effect on body weight was due to the reduced digestibility of amylose and thereby lower dietary ME content. Overall, this confirmed that Dodamssal rice had a positive effect on weight loss in dogs, and these results suggest that Dodamssal rice has potential value as a raw ingredient for preventing obesity in dogs.

## 1. Introduction

Globally, the incidence of obesity and related diseases is increasing rapidly, not only in humans but also in companion animals, having emerged as a major clinical problem in modern veterinary medicine [1,2]. Overweight is generally defined as up to 20% above ideal weight, obese >20%; however, several reports have specified that if dogs weigh 15% [3], 20% [4], or 30% [5] more than their ideal weight, they can be considered over-weight or obese. It is accepted that obesity is primarily caused by excessive energy intake, or decreased energy expenditure [3]. Further, obesity is associated with the onset or worsening of various clinical diseases, such as dyslipidemia, insulin resistance, metabolic syndrome, endocrine disorders (such as diabetes mellitus or hypothyroidism), orthopedic disorders (including osteoarthritis and intervertebral disc disease), and neoplasia [6]. Accordingly, interest in the prevention and treatment of dog obesity is steadily increasing, since these afflictions not only worsen the quality of life for the dogs themselves, but also increase the financial burden on dog owners [7].

All grains, such as wheat, corn, and rice, contain starch, and are a major source of carbohydrates in both human and pet diets. Starch is chemically comprises amylopectin, composed of α-D-(1-4) and α-D-(1-6) glycosidic linkages, and amylose, composed of α-D-(1-4) glycosidic linkages [8]. Since amylopectin (branched chain structure) is more sensitive to the action of digestive enzymes than amylose (linear structure), their ratio in starch sources may affect digestion rates [9]. Accordingly, starch types can be classified into rapidly digestible starch (RDS), slowly digestible starch (SDS), and resistant starch (RS) depending on the ratio of amylopectin to amylose [10]. Although RDS and SDS have different digestion rates, they are mostly digested in the small intestine, whereas RS is not digested by digestive enzymes but is digested by microbial enzymes [11]. Notably, prior studies have shown a positive correlation between the content of amylose in starch and the formation of RS [12,13,14,15]. Moreover, RS has fibrous-like physiological properties [16,17], and promising correlated benefits in humans and animals (such as reduced blood glucose, cholesterol, and body fat; enhanced prebiotic fiber effects; and improved mineral absorption), while it has also been reported to bolster disease prevention (including colonic diseases and inflammatory bowel disease) [18,19,20,21,22,23,24,25,26,27]. In the past, the metabolic effects and potential health benefits of grains containing native RS have been studied extensively; however, previous researchers have focused on digestive absorption or functional properties in dogs [28,29,30], while few studies have evaluated its effect on weight loss or nutritional safety.

Grains are an important carbohydrate source in dog diets, with most extruded pet foods containing 20–50% carbohydrates [31]. Nutritionally, carbohydrates are not essential nutrients [32]; yet, when compared to animal protein or fats, carbohydrates are an extremely economical energy source for use in pet foods [33]. Although grains are a widely used carbohydrate source within the pet food industry, many companion animal owners are concerned about feeding their pets grains, and studies on the safety of carbohydrate sources available in dog foods are limited [34]. 

Dodamssal (*Oryza sativa* L.) is a novel variety of rice developed by the National Institute of Food Science and Technology in Korea, specifically designed with high amylose and RS contents [35]. Thus, this study was conducted to investigate the effects of Dodamssal rice on weight loss and physiological properties in companion dogs.

## 2. Materials and Methods

### 2.1. Animals, Management, and Housing

This experiment was conducted in accordance with the method approved by the animal care and use committee, National Institute of Animal Science (NIAS-2017-255). Each dog was housed in an individual room (1.7 m × 2.1 m), held at a consistent temperature (22–24 °C), and with consistent lighting cycles (12 h light, 12 h dark) during the experiment period. All dogs were provided approximately three hours of outdoor activity every day with other dogs for socialization. Food was provided at an amount estimated by the maintenance energy requirement (MER) equation for each dog twice per day throughout the duration of the experiment, while drinking water was provided ad libitum. The MERs (kcal/day) of the dogs were calculated by considering the MER equation suggested by association of American feed control officials (AAFCO) [36] and MER of inactive adult dogs as follows: MER = 102 kcal × metabolic body weight (mBW, kg; BW^0.75^). Food intake was measured daily, and body weight (BW) and body condition score (BCS) were measured weekly using a 9-point scale. The rate of change in body weight gain (BWG) was calculated using the measured body weight.

### 2.2. Experimental Design

#### 2.2.1. Experiment 1

A total of 24 spayed female small-breed dogs (7.60 ± 0.01 year-old; initial BW 4.73 ± 0.31 kg; 9 Poodles, 6 Maltese, and 9 miniature Schnauzers,) were used in Experiment 1 (Exp. 1). The dogs were randomly divided into three groups (*n* = 8 dogs per group): Group 1 was fed a normal-fat basal diet (Control), Group 2 was fed a high-fat diet with 12% normal amylose type rice (hNAR; Shindongjin rice, Korea), and Group 3 was fed a high-fat diet with 12% high amylose type rice (hHAR; Dodamssal rice, Korea). The formulation and chemical composition of experimental diets in Exp. 1 are presented in Table 1.

#### 2.2.2. Experiment 2

A total of 8 spayed female small-breed dogs (9.60 ± 0.01 year-old; initial BW 4.28 ± 0.63 kg; 2 Schnauzers, 2 Maltese, 2 Yorkshire Terriers, 1 Shih Tzu, and 1 Pomeranian) were used in Experiment 2 (Exp. 2). The dogs were randomly divided into two groups (*n* = 4 dogs per group): Group 1 was fed a basal diet with 28.76% normal amylose type rice (NAR; Shindongjin rice, Korea), and Group 2 was fed a basal diet with 28.76% high amylose type rice (HAR; Dodamssal rice, Korea). The experiment comprised consistent diets fed over 4 weeks. The formulation and chemical composition of experimental diets in Exp. 2 are presented in Table 2.

### 2.3. Preparation of Experimental Diets

Shindongjin rice was purchased from the market, whereas Dodamssal rice flour was supplied from the National Institute of Agricultural Sciences (Wanju, Republic of Korea). The basal diet was formulated to meet the nutritional requirements for adult dogs, as suggested by the AAFCO [36]. The high-fat diet was prepared by adding lard to exceed the metabolizable energy of the basal diet by approximately 20% (Table 1 and Table 2), and all experimental diets were prepared following the procedure previously reported in Seo et al. [37]. No palatants were used in the experimental diets. The experimental diets were stored at –20 °C until feeding and thawed at room temperature (RT) until reaching RT 1 day before the feeding test.

### 2.4. Sampling and Analysis

The chemical compositional parameters of the experimental diets were analyzed following the standard association of official analytical chemist methods (AOAC) [38], including the moisture content (AOAC method 934.01), crude protein (CP, AOAC method 984.13), ether extract (EE, AOAC method 920.39), crude ash (CA, AOAC method 942.05), crude fiber (CF, AOAC method 978.10), calcium (Ca, AOAC method 927.02), and phosphorus (P, AOAC method 965.17). The nitrogen-free extract (NFE) was calculated according to Equation (1): The ME in experimental diets was calculated according to Equation (2)

NFE (%) = 100 − (Moisture + CP + CF + EE + CA)(1)

ME, metabolizable energy (kcal/kg) = ((CP × 3.5) + (EE × 8.5) + (NFE × 3.5)) × 10(2)

Blood samples were collected from the jugular vein after 12 h of fasting, every 4 weeks for both Exp. 1 and 2. The collected blood was immediately separated into EDTA vacutainer tubes (ref 367861, BD Vacutainer, NJ, USA) and serum vacutainer tubes (ref 367812, BD Vacutainer, NJ, USA). Whole blood in the EDTA vacutainer tubes was used for complete blood cell count (CBC) analysis immediately after collection. The serum was obtained by centrifugation (2000× *g*, 10 min) from blood in the serum vacutainer tubes, and then stored frozen (−80 °C) until analysis. CBCs were measured using an automatic hematology analyzer (IDEXX Laboratories, Inc., Westbrook, ME, USA), and serum biochemical parameters were analyzed using an automatic biochemical analyzer (Hitachi 7180; Hitachi High-Technologies Co., Tokyo, Japan). 

### 2.5. Statistical Analysis

All statistical analyses were performed using SPSS (*v*.17.0; SPSS Statistics, IL, USA, 2009), and all data are presented as means ± standard error. The significant differences among treatment groups in Exp. 1 were analyzed by univariate analysis via a general linear model with Tukey test as the post-hoc analysis. The significant differences between NAR and HAR groups in Exp. 2 were analyzed using Student’s *t*-test, and all differences were considered statistically significant if *p* < 0.05.

## 3. Results

### 3.1. Experiment 1

#### 3.1.1. Food Intake and Body Parameters 

Table 3 shows the changes in feed intake and body parameters by feeding of high-fat diet and/or high-resistant starch. During the 24 weeks, no significant differences in average daily feed intake (ADFI), BW, and BCS were observed among all experimental groups (*p* > 0.05). ME intake and calculated ME intake per kg mBW tended to be lower in the control group than in the hNAR and hHAR groups, but no statistically significant difference was observed (*p* > 0.05). BWG and rate of gain were significantly increased in hNAR and hHAR groups compared with the control (*p* < 0.01); however, no significant differences were observed between the hNAR and hHAR groups (*p* > 0.05). 

#### 3.1.2. Hematological and Biochemical Parameters

Table 4 shows the results of hematological parameters analyzed from blood collected at the end of Exp. 1. Notably, all parameters of the three groups were within the normal reference range, and no significant differences were observed among any of the groups.

**Figure 1:**
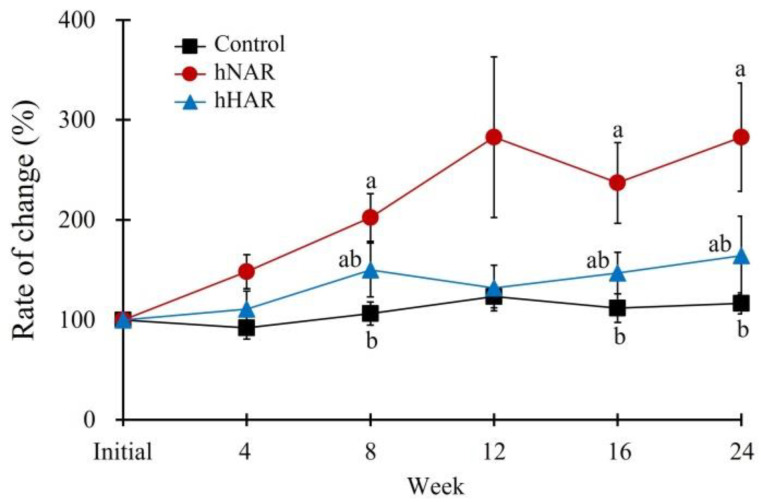
Effect of high-fat diet with high amylose type rice on the change in serum ALT levels in dogs (Exp. 1). Data are expressed as percent change compared to the initial value in each group. Control group, basal diet with 12% normal amylose type rice. hNAR group, high-fat diet with 12% normal amylose type rice. hHAR group, high-fat diet with 12% high amylose type rice. The different letters in same week denote significant differences among each group (*p* < 0.05).

Table 5 shows the serum biochemical parameters at the end of the Exp. 1. The parameters in all experimental groups except serum alanine aminotransferase (ALT) in the hNAR group were within the reference range, and no significant differences were recorded among the three groups for serum glucose (GLU), creatinine (CREA), blood urea nitrogen (BUN), phosphorous (PHOS), total protein (T-PRO), albumin (ALB), and total bilirubin (T-BIL) levels (*p* > 0.05). Serum total cholesterol (T-CHO) levels were significantly higher in the hHAR group compared to the control (*p* < 0.05), although no significant difference was observed compared to the hNAR group (*p* > 0.05). The ALT, a notable indicator of liver or hepatobiliary function, showed a tendency to be higher in the hNAR group than that of the control and hHAR groups at the end of the experiment (*p* = 0.090). On the other hand, the ALT change rate over time during the experimental period significantly increased in the hNAR group compared with that of the control (*p* < 0.05), while the hHAR group did not significantly increase compared with that of the control (Figure 1).

### 3.2. Experiment 2

#### 3.2.1. Feed Intake and Body Parameters 

Exp. 2 was conducted to investigate whether high amylose type rice could induce weight loss in dogs. Table 6 shows the feed intake and body parameters by feeding of high amylose type rice. During the experimental period, there was no significant difference in ADFI, BW, and BCS between the two experimental groups (*p* > 0.05); however, BWG was decreased in the HAR group compared to the NAR group (*p* < 0.05). The rate of weight gain tended to decrease in the HAR group compared to NAR group, although this difference was not statistically significant (*p* = 0.094).

#### 3.2.2. Hematological and Biochemical Parameters

Table 7 shows the hematological parameters at the end of the Exp. 2. All parameters of both experimental groups were within the normal reference range, and no significant differences were observed between the two groups.

The serum biochemical parameters are presented in Table 8. Similarly, all parameters analyzed in Exp.2 were within the normal reference ranges, and there were no significant differences between the NAR and HAR groups.

## 4. Discussion

### 4.1. Body Parameters and Feeding

Previous animal studies evaluating the effects of RS supplements have reported that the level of high-dose RS not only lowers fat weight [39,40], but also decreases the size of adipocytes [41]. Furthermore, some human studies have suggested that the supply of RS may contribute to the prevention of excessive adipose tissue accumulation over the long term by promoting lipid acidification in vivo [24]. Therefore, it was expected that a high RS with high amylose content diet could suppress weight gain in dogs, despite the dogs being fed a high-fat diet. However, in the results of Exp. 1, when a high-fat diet containing 12% high amylose type rice (Dodamssal rice) was provided for 24 weeks, no significant differences in BW, BWG and rate of gain values were observed between hNAR and hHAR groups. The BW and BWG in both groups of hNAR and hHAR were higher than the control group. This is consistent with the results of Polakof et al. [42], where no change in BW was reported when rodents were fed a high-fat diet containing RS for 9 weeks. Meanwhile, Xu et al. [43] and Si et al. [44] found that high-fat diet induced obese rodents provided with RS led to weight loss. In Exp. 1, common cooked rice was partially used as a carbohydrate source across all three treatment groups (Control, 20%; hNAR and hHAR, 18.7%), as a part of the carbohydrate feedstock was filled by normal amylose type rice and high amylose type rice. Therefore, the fact that the hHAR-fed group did not show a lower weight-gain effect compared with the hNAR-fed group in Exp. 1 could be due to an insufficient content of high amylose type rice used in the experimental diet to counteract the weight gain induced by a high-fat diet.

Exp. 2 investigated the effects of using only the NAR or HAR as a carbohydrate source on BW in both groups. The results showed that the weight gain in dogs fed on a high amylose type rice-based diet for 4 weeks was lower than that in the NAR group, thereby supporting the hypothesis that animals fed a high-RS-containing feed experienced reduced BW [43,44]. Resistant starch has very low digestibility and will therefore reduce the energy content of the diet. The effect on weight loss is considered to be due to the lower dietary ME by reduced digestibility. In addition, this finding is consistent with the results of Lerer-Metzger et al. [45] where, after providing a basic feed containing 57% mung bean (*Phaseolus aureus*) starch group (RS, 77 g/kg) for 5 weeks to rodents, a lower weight gain was observed than with a wheat starch group (RS, 22 g/kg).

The results of the present study showed that a high amylose type rice-based diet could not suppress the weight gain caused by the intake of excessive fat (> 15%, DM basis); however, in the case of a high amylose type rice-based diet containing a normal level of fat (9%, DM basis), it had an observable effect on weight loss in dogs. Although no nutrient digestibility of the HAR-based diet was evaluated in this study, RS is well known to be characterized by low or no degradation from digestive enzymes [14,15]. Accordingly, it was considered that the weight loss observed in Exp. 2 was due to a decrease in nutrient digestibility. Moreover, the HAR-based diet did not affect feed intake, and it suggests that these results did not negatively affect palatability in both Exp. 1 and 2.

### 4.2. Safety and Blood Parameters

Hematological parameters provide information on pre-existent anemia, possible infections, inflammatory response, immune function, or stress [46]. Thus, these parameters are used as significant indicators to evaluate the feed safety [36]. Some studies in humans [47] and dogs [48] have demonstrated the increase in WBC, NEU, PLT, and LYM in chronically obese patients. These changes appear to be significantly related to low-grade chronic inflammation, which is predominantly observed in patients with chronic obesity, although the underlying mechanism has yet to be fully elucidated [48,49,50]. However, in the present study, no significant changes of hematological parameters were observed in dogs fed high-fat diets (Exp. 1), nor for the intake of HAR (Exp. 1 and 2). All parameters were maintained within reference ranges, indicating that HAR can be used as an ingredient for pet food without compromising the health and safety of dogs.

Biochemical parameters were measured to observe the effects of high-fat or regular basal diets containing HAR on the dogs’ hepatic functions, kidney filtration capacity, other internal organ tasks, or metabolic profiles [46]. Several studies have reported the differences in the levels of some biochemical parameters (for example, T-CHO, ALT, aspartate aminotransferase, alkaline phosphatase, and gamma glutamyl transferase) in overweight or obese dogs compared to normal-weight dogs [51,52,53,54]. Notably, these changes were consistent with the results of the present study, showing an increase in serum T-CHO and ALT levels when dogs were fed high-fat diets for 24 weeks (Exp. 1). Increases in lipid profiles, such as cholesterol, triglycerides, and others, are known to derive from changes in lifestyle or increases in energy or feed intake [51,55], while hyperlipidemia and hypercholesterolemia are reported to be common findings in overweight and obese dogs [56]. ALT, also known as glutamic-pyruvic transaminase (GPT), is mainly present in liver cells, and is generally used as an indicator to measure hepatocellular injury, as it is released into the blood by damage such as hepatocyte necrosis, cell membrane rupture, and hepatocyte degeneration [57,58]. In addition, ALT increases have been shown to strongly correlate with the accumulation of fat in the liver [59,60,61]. Therefore, in the present study, the increases in serum T-CHO and ALT were considered to be caused by high-fat diets (Exp. 1). Moreover, it is assumed that the increase in serum ALT levels might be related to the accumulation of fat in the liver tissue. Prior obesity studies in rodents have reported that RS supplementation can effectively reduce levels of triglycerides and low-density lipoprotein cholesterol (LDL-C), as well as serum T-CHO [43,44,62,63]. Although the highest levels of serum T-CHO was observed in the hHAR group, there was no significant difference between hNAR and hHAR (Exp. 1). The inconsistencies between the present study and previous studies are thought to be due to the different levels of RS in the diet. It is considered that the dietary RS content in this study may not have been sufficient to reduce the serum T-CHO level bolstered by an excessively high-fat diet; therefore, additional studies are necessary to identify the effective level and mechanisms of RS on cholesterol reduction.

Notably, the change rates of ALT level in the hNAR group increased gradually throughout the experiment, whereas the hHAR group showed a similar tendency to the control group (Exp. 1). These results were consistent with several studies of obesity in rodents [44]. Wang et al. [62], Martinez et al. [63], and Xu et al. [43] demonstrated a decrease in ALT levels and adipose tissues in the liver as a result of feeding RS to obese rodents, in addition to a close relationship between increasing ALT and obesity, and the formation of adipose tissue in the liver. Although this study showed the effect of HAR feeding on reduction of serum ALT level in dogs fed a high-fat diet, it is difficult to conclude that HAR has a positive effect on liver function. Thus, additional studies are needed in a dog model with liver dysfunction or fatty liver to verify the effect of HAR in dogs.

## 5. Conclusions

This study was conducted to investigate the safety and anti-obesity effect of Dodamssal rice (high amylose type rice) as a raw ingredient for dog food. Our results showed the potential ability of a normal diet containing Dodamssal rice to reduce BWG in adult dogs. The effect on BW was because of reduced digestibility of amylose and thereby lower dietary ME content. In addition, we confirmed that all hematological parameters to evaluate safety of feed ingredients were within normal reference range for healthy dogs. This suggests that the Dodam rice, which has a remarkably high RS content, is not only safe as a raw ingredient for dog food, but also has potential value as a functional ingredient for preventing obesity. From a veterinary point of view, however, additional studies are needed in dog models with preexisting obesity or liver dysfunctions such as fatty liver.

## Figures and Tables

**Table 1:** Formulation and chemical composition of experimental diets (Exp. 1).

Items	Ingredients		Basal Diet	High-Fat Diet	
	NAR ^1^	HAR ^2^	Control ^3^	hNAR ^4^	hHAR ^5^
Ingredients, %					
Chicken breast powder	-	-	12	17	17
Egg yolk powder	-	-	12	12	12
Anchovy powder	-	-	0.5	0.5	0.5
Rice flour (Normal amylose type)	-	-	12	12	-
Rice flour (High amylose type)	-	-	-	-	12
Sweet potato powder	-	-	10	-	-
Lard	-	-	-	8	8
Cooked rice (Normal amylose type)	-	-	20	18.7	18.7
Egg-shell powder	-	-	1	1	1
Potassium Gluconate	-	-	1	1.2	1.2
Vitamin-Mineral premix ^6^	-	-	0.5	0.6	0.6
Water	-	-	31	29	29
Chemical compositions, % (DM basis, analyzed)					
Dry matter (DM)	86.9	86.7	53.0	56.2	55.6
Crude protein (CP)	6.67	7.57	32.6	36.8	37.1
Ether extract (EE)	0.43	1.46	16.4	27.9	29.5
Crude fiber (CF)	0.03	0.18	1.0	0.5	0.7
Crude ash (CA)	0.55	0.68	3.8	3.6	3.7
Nitrogen-free extract (NFE)	92.1	89.7	51.5	34.0	34.4
Calcium (Ca)	0.08	0.09	0.8	0.7	0.8
Phosphorus (P)	0.15	0.21	0.5	0.5	0.5
Resistant starch (RS)	2.68	15.57	1.4	1.3	7.5
ME, kcal/kg (calculated)	-	-	4155	4748	4816

Values of chemical composition are analyzed data as wet based except for ME (DM based, calculated). ^1^ NAR, normal amylose type rice (Shindongjin rice). ^2^ HAR, high amylose type rice (Dodamssal rice). ^3^ Control group, basal diet with 12% NAR. ^4^ hNAR group, high-fat diet with 12% NAR. ^5^ hHAR group, high-fat diet with 12% HAR. ^6^ Provided per kilogram of diet: Vit. A, 5250 IU; Vit. D3, 375 IU; Vit. E, 37.5 mg; Vit. K, 0.078 mg; Vit. B1 (thiamine), 4.2 mg; Vit. B2 (riboflavin), 3.9 mg; Vit. B6 (pyridoxine), 3 mg; Vit. B12, 0.021 mg; Cal. D. Pantothenate, 9 mg; Niacin, 45 mg; Folic acid, 0.6 mg; Biotin, 0.054 mg; Taurine, 1500 mg; FeSO_4_ H_2_O, 66 mg; MnSO_4_ H_2_O, 5.7 mg; ZnSO_4_ H_2_O, 75 mg; CuSO_4_ H_2_O, 11.25 mg; Na_2_SeO_3_, 0.27 mg; Ca_2_ (IO_3_), 1.35 mg. ME, metabolizable energy (kcal/kg) = ((CP × 3.5) + (EE × 8.5) + (NFE × 3.5)) × 10.

**Table 2:** Formulation and chemical composition of experimental diets (Exp. 2).

Items	NAR ^1^	HAR ^2^
Ingredients, %		
Chicken breast powder	12.7	12.7
Egg yolk powder	12	12
Rice flour (Normal amylose type)	28.76	-
Rice flour (High amylose type)	-	28.76
Lard	2.9	2.9
Green laver powder	1	1
Calcium carbonate	1.04	1.04
Potassium Citrate	0.6	0.6
Monocalcium phosphate	0.4	0.4
Vitamin-Mineral premix ^3^	0.4	0.4
Salt (NaCl)	0.2	0.2
Water	40	40
Chemical compositions, % (DM basis, analyzed)		
Dry matter (DM)	55.7	52.8
Crude protein (CP)	33.9	33.1
Ether extract (EE)	16.9	17.4
Crude fiber (CF)	0.2	0.7
Crude ash (CA)	4.9	4.6
Nitrogen-free extract (NFE)	44.2	44.1
Ca	0.9	0.9
P	0.6	0.6
ME, kcal/kg (DM basis, calculated)	4165	4184

Values of chemical composition are analyzed data as wet based except for ME (DM based, calculated). ^1^ NAR, basal diet with 28.76% NAR. ^2^ HAR group, basal diet with 28.76% HAR. ^3^ Provided per kilogram of diet: Vit. A, 3500 IU; Vit. D3, 250 IU; Vit. E, 25 mg; Vit. K, 0.052 mg; Vit. B1 (thiamine), 2.8 mg; Vit. B2 (riboflavin), 2.6 mg; Vit. B6 (pyridoxine), 2 mg; Vit. B12, 0.014 mg; Cal. D. Pantothenate, 6 mg; Niacin, 30 mg; Folic acid, 0.4 mg; Biotin, 0.036 mg; Taurine, 1000 mg; FeSO_4_ H_2_O, 44 mg; MnSO_4_ H_2_O, 3.8 mg; ZnSO_4_ H_2_O, 50 mg; CuSO_4_ H_2_O, 7.5 mg; Na_2_SeO_3_, 0.18 mg; Ca_2_ (IO_3_), 0.9 mg. ME, metabolizable energy (kcal/kg) = ((CP × 3.5) + (EE × 8.5) + (NFE × 3.5)) × 10.

**Table 3:** Effects of high-fat diets with high amylose type rice on feed intake and body parameters in dogs (Exp. 1).

Items	Normal Diet			High-Fat Diet						*p*-Value
	Control ^1^			hNAR ^2^			hHAR ^3^			
ADFI (g/d)	151	±	11	146	±	10	141	±	13	0.850
MER (kcal/d)	330	±	22	332	±	21	319	±	26	0.946
MEI (kcal/d)	332	±	23	389	±	28	378	±	33	0.342
Calculated MEI/kg mBW	103	±	5	119	±	5	121	±	17	0.304
Body weight (kg)										
Initial	4.8	±	0.6	4.8	±	0.5	4.6	±	0.6	0.946
Final	4.7	±	0.6	5.5	±	0.6	5.3	±	0.6	0.618
*p*-value ^4^	0.913			0.416			0.382			
BWG (kg)	−0.1	±	0.1 ^b^	0.9	±	0.3 ^a^	0.7	±	0.1 ^a^	0.006
Rate of gain (%)	−1.8	±	2.9 ^b^	21	±	9 ^a^	17	±	3 ^a^	0.009
Body condition score										
Initial	5.0	±	0.3	4.8	±	0.3	4.8	±	0.3	0.794
Final	5.0	±	0.3	5.3	±	0.3	5.1	±	0.3	0.853
*p*-vlaue ^4^	0.989			0.278			0.349			

^1^ Control group, basal diet with 12% normal amylose type rice. ^2^ hNAR group, high-fat diet with 12% normal amylose type rice. ^3^ hHAR group, high-fat diet with 12% high amylose type rice. ^4^ *p*-values for comparisons between the initial and final values in the same column. ADFI, average daily food intake; MER, metabolic energy requirement, MEI, metabolic energy intake; mBW, metabolic body weight; BWG, body weight gain. ^a,b^ Data without same superscript in a same row differ significantly (*p* < 0.05).

**Table 4:** Effects of high-fat diets with high amylose type rice on hematological parameters in dogs (Exp. 1).

Items	Normal Diet			High-Fat Diet						*p*-Value
	Control ^1^			hNAR ^2^			hHAR ^3^			
Leukocytes										
WBC (×10^6^/mL) (Ref. range: 5.05–16.76)	7.1	±	1.2	8	±	0.9	8.8	±	1.3	0.590
NEU (×10^6^/mL) (Ref. range: 2.95–11.64)	4.8	±	0.9	5.5	±	0.6	5.8	±	0.9	0.718
LYM (×10^6^/mL) (Ref. range: 1.05–5.10)	1.6	±	0.1	1.8	±	0.3	2.1	±	0.3	0.394
MONO (×10^6^/mL) (Ref. range: 0.16–1.12)	0.4	±	0.1	0.4	±	0.1	0.5	±	0.1	0.815
EOS (×10^6^/mL) (Ref. range: 0.06–1.23)	0.2	±	0.1	0.3	±	0.1	0.5	±	0.1	0.221
BASO (×10^6^/mL) (Ref. range: 0–0.1)	0.01	±	0.00	0.01	±	0.00	0.01	±	0.00	0.269
Erythrocytes										
RBC (×10^9^/mL) (Ref. range: 5.65–8.87)	6.8	±	0.2	6.8	±	0.1	6.9	±	0.2	0.958
HGB (g/dL) (Ref. range: 13.1–20.5)	15	±	0.5	16	±	0.3	15	±	0.4	0.697
HCT (%) (Ref. range: 37.3–61.7)	43	±	1.6	46	±	1.1	45	±	1.1	0.447
MCH (pg) (Ref. range: 21.2–25.9)	22	±	0.3	23	±	0.3	23	±	0.4	0.170
Thrombocytes										
PLT (K/µL) (Ref. range: 148–484)	284	±	39	330	±	63	266	±	37	0.624
PCT (%) (Ref. range: 0.14–0.46)	0.4	±	0.0	0.4	±	0.1	0.4	±	0.0	0.476

^1^ Control group, basal diet with 12% normal amylose type rice. ^2^ hNAR group, high-fat diet with 12% normal amylose type rice. ^3^ hHAR group, high-fat diet with 12% high amylose type rice. WBC, white blood cell; NEU, neutrophils; LYM, lymphocytes; MONO, monocytes; EOS, eosinophils; BASO, basophils; RBC, red blood cells; HGB, hemoglobin; HCT, hematocrit; MCH, mean corpuscular hemoglobin; PLT, platelet; PCT, plateletcrit.

**Table 5:** Effects of high-fat diets with high amylose type rice on serum biochemical parameters in dogs (Exp. 1).

Items	Normal Diet			High-Fat Diet						*p*-Value
	Control ^1^			hNAR ^2^			hHAR ^3^			
GLU (mg/dL) (Ref. range: 70–138)	89	±	3.4	98	±	8.0	97	±	4.7	0.480
CREA (mg/dL) (Ref. range: 0.5–1.6)	0.6	±	0.1	0.6	±	0.03	0.6	±	0.1	0.676
BUN (mg/dL) (Ref. range: 6.0–31)	16	±	1.3	13	±	0.6	15	±	0.9	0.136
PHOS (mg/dL) (Ref. range: 2.5–6.0)	4.3	±	0.2	4.3	±	0.1	4.4	±	0.3	0.873
T-Pro (g/dL) (Ref. range: 5.0–7.4)	6.6	±	0.1	6.6	±	0.2	6.7	±	0.1	0.679
ALB (g/dL) (Ref. range: 2.7–4.4)	3.2	±	0.1	3.2	±	0.2	3.2	±	0.1	0.965
ALT (U/L) (Ref. range: 12–118)	71	±	11	143	±	26	101	±	24	0.090
T-BIL (mg/dL) (Ref. range: 0.1–0.3)	0.2	±	0.02	0.2	±	0.04	0.3	±	0.03	0.247
T-CHO (mg/dL) (Ref. range: 29–291)	155	±	19 ^b^	192	±	9 ^ab^	231	±	20 ^a^	0.015

^1^ Control group, basal diet with 12% normal amylose type rice. ^2^ hNAR group, high-fat diet with 12% normal amylose type rice. ^3^ hHAR group, high-fat diet with 12% high amylose type rice. GLU, glucose; CREA, creatinine; BUN, blood urea nitrogen; PHOS, phosphorous; T-Pro, total protein; ALB, albumin; ALT, alanine aminotransferase; T-BIL, total bilirubin; T-CHO, total cholesterol. ^a,b^ Data without same superscript in a same row differ significantly (*p* < 0.05).

**Table 6:** Effects of high amylose type rice on feed intake and body parameters in dogs (Exp. 2).

Items	NAR ^1^			HAR ^2^			*p*-Value
ADFI (g/d)	146	±	28	150	±	22	0.909
MER (kcal/d)	305	±	53	302	±	44	0.972
MEI (kcal/d)	339	±	71	332	±	66	0.936
Calculated MEI/kg mBW	113	±	8	112	±	7	0.908
Body weight (kg)							
Initial	4.3	±	1.1	4.3	±	0.9	0.972
Final	4.2	±	1.1	4.0	±	0.8	0.872
*p*-vlaue ^3^	0.952			0.821			
BWG (kg)	−0.1	±	0.00	−0.3	±	0.1	0.032
Rate of gain (%)	−3.0	±	1.0	−6.7	±	1.5	0.094
Body condition score							
Initial	4.5	±	0.3	4.5	±	0.5	0.998
Final	4.3	±	0.3	4.0	±	0.4	0.620
*p*-vlaue ^3^	0.537			0.468			

^1^ NAR group, normal amylose type rice-based diet. ^2^ HAR group, high amylose type rice-based diet. ADFI, average daily feed intake; MER, metabolic energy requirement, MEI, metabolic energy intake; mBW, metabolic body weight; BWG, body weight gain. ^3^ *p*-values for comparisons between the initial and final values in the same column.

**Table 7:** Effects of high amylose type rice on hematological parameters in dogs (Exp. 2).

Items	NAR ^1^			HAR ^2^			*p*-Value
Leukocytes							
WBC (×10^6^/mL) (Ref. range: 5.05–16.76)	8.2	±	0.8	6.6	±	0.7	0.206
NEU (×10^6^/mL) (Ref. range: 2.95–11.64)	4.6	±	0.5	3.8	±	0.5	0.309
LYM (×10^6^/mL) (Ref. range: 1.05–5.10)	2.7	±	0.6	2.2	±	0.3	0.393
MONO (×10^6^/mL) (Ref. range: 0.16–1.12)	0.4	±	0.1	0.3	±	0.1	0.639
EOS (×10^6^/mL) (Ref. range: 0.06–1.23)	0.5	±	0.1	0.3	±	0.1	0.256
BASO (×10^6^/mL) (Ref. range: 0–0.1)	0.01	±	0.01	0.00	±	0.00	0.356
Erythrocytes							
RBC (×10^9^/mL) (Ref. range: 5.65–8.87)	6.7	±	0.2	6.9	±	0.3	0.602
HGB (g/dL) (Ref. range: 13.1–20.5)	16	±	0.4	17	±	0.7	0.680
HCT (%) (Ref. range: 37.3–61.7)	46	±	0.7	49	±	2.2	0.316
MCH (pg) (Ref. range: 21.2–25.9)	24	±	0.6	24	±	0.4	0.735
Thrombocytes							
PLT (K/µL) (Ref. range: 148–484)	483	±	85	403	±	62	0.478
PCT (%) (Ref. range: 0.14–0.46)	0.4	±	0.1	0.4	±	0.1	0.575

^1^ NAR group, normal amylose type rice-based diet. ^2^ HAR group, high amylose type rice-based diet. WBC, white blood cell; NEU, neutrophils; LYM, lymphocytes; MONO, monocytes; EOS, eosinophils; BASO, basophils; RBC, red blood cells; HGB, hemoglobin; HCT, hematocrit; MCH, mean corpuscular hemoglobin; PLT, platelet; PCT, plateletcrit.

**Table 8:** Effects of high amylose type rice on serum biochemical parameters in dogs (Exp. 2).

Items	NAR ^1^			HAR ^2^			*p*-Value
GLU (mg/dL) (Ref. range: 70–138)	107	±	4	101	±	2	0.196
CREA (mg/dL) (Ref. range: 0.5–1.6)	0.8	±	0.1	0.9	±	0.04	0.122
BUN (mg/dL) (Ref. range: 6.0–31)	18	±	1	17	±	1	0.568
PHOS (mg/dL) (Ref. range: 2.5–6.0)	3.4	±	0.1	3.6	±	0.4	0.618
T-Pro (g/dL) (Ref. range: 5.0–7.4)	6.5	±	0.2	6.7	±	0.4	0.697
ALB (g/dL) (Ref. range: 2.7–4.4)	2.7	±	0.1	2.8	±	0.1	0.303
ALT (U/L) (Ref. range: 12–118)	46	±	8	39	±	8	0.545
T-BIL (mg/dL) (Ref. range: 0.1–0.3)	0.03	±	0.01	0.05	±	0.01	0.134
T-CHO (mg/dL) (Ref. range: 29–291)	220	±	32	168	±	19	0.208
TG (mg/dL) (Ref. range: 23–102)	44	±	1	27	±	8	0.273
NEFA, mEq/L (Ref. range: 0.13–1.25)	0.8	±	0.1	0.6	±	0.1	0.328

^1^ NAR group, normal amylose type rice-based diet. ^2^ HAR group, high amylose type rice-based diet. GLU, glucose; CREA, creatinine; BUN, blood urea nitrogen; PHOS, phosphorous; T-Pro, total protein; ALB, albumin; ALT, alanine aminotransferase; T-BIL, total bilirubin; T-CHO, total cholesterol; TG, triglycerides; NEFA, nonesterified fatty acids.

## Data Availability

Not applicable.
